# Acute Exacerbations of Interstitial Lung Diseases: Focus on Biomarkers

**DOI:** 10.3390/ijms241210196

**Published:** 2023-06-15

**Authors:** Fotios Drakopanagiotakis, Philipp Markart, Paschalis Steiropoulos

**Affiliations:** 1 Department of Respiratory Medicine, Medical School, Democritus University, 68100 Alexandroupolis, Greece; fdrakopanagiotakis@gmail.com; 2 Department of Respiratory Medicine, Klinikum Fulda and University Medicine Campus Fulda, Pacelliallee 4, 36043 Fulda, Germany; philipp.markart@klinikum-fulda.de

**Keywords:** interstitial lung diseases, acute exacerbations, biomarkers

## Abstract

Interstitial lung diseases (ILDs) are a large group of pulmonary disorders characterized histologically by the cardinal involvement of the pulmonary interstitium. The prototype of ILDs is idiopathic pulmonary fibrosis (IPF), an incurable disease characterized by progressive distortion and loss of normal lung architecture through unchecked collagen deposition. Acute exacerbations are dramatic events during the clinical course of ILDs, associated with high morbidity and mortality. Infections, microaspiration, and advanced lung disease might be involved in the pathogenesis of acute exacerbations. Despite clinical scores, the prediction of the onset and outcome of acute exacerbations is still inaccurate. Biomarkers are necessary to characterize acute exacerbations better. We review the evidence for alveolar epithelial cell, fibropoliferation, and immunity molecules as potential biomarkers for acute exacerbations of interstitial lung disease.

## 1. Introduction

Interstitial lung diseases (ILDs) are pulmonary disorders characterized histologically by the involvement of the pulmonary interstitium. Clinical manifestations of these include dyspnea, coughing, hypoxia, and impaired lung function. Several of these conditions are associated with underlying systemic conditions, such as connective tissue diseases (CTDs), asbestos exposure, drug exposure, or allergen exposure, as in hypersensitivity pneumonitis. It is nevertheless unclear in many cases what causes ILDs [1]. Idiopathic pulmonary fibrosis (IPF) is the prototype of ILDs. This incurable disease is characterized by progressive distortion of the lung architecture, resulting in the formation of honeycomb cysts, traction bronchiectasis, and reticulation through unchecked collagen deposition. Diagnostic criteria usually require a combination of imaging, clinical, and laboratory findings [2]. In many cases, however, non-invasive measures are insufficient to diagnose an ILD accurately. An invasive procedure such as a surgical lung biopsy or a bronchoscopic cryo-biopsy may be required for such patients. Although this approach can be feasible in a small number of patients, comorbidities, severe functional impairment, and aged patients limit the applicability of this approach [2].

Patients with ILDs have unpredictable clinical outcomes. There is often a slow but steady progression of the disease in many patients. In some patients, the ILD remains stable, while other patients experience a rapid clinical deterioration. Acute worsening of the ILD (known as acute exacerbation) can also occur. Acute exacerbations may be fatal or may lead to significant worsening of symptoms [1,2].

Acute exacerbations of interstitial lung diseases (AE-ILDs) refer to a sudden aggravation of symptoms in patients with ILDs. The exacerbations of chronically present ILD were initially recognized in IPF, but they can occur in almost all ILDs. These exacerbations are characterized by increased dyspnea and cough and decreased oxygen saturation. Acute exacerbation of interstitial lung diseases (AE-ILD) is an acute and often morbid deterioration of the patient’s respiratory function leading to hospitalization.

The Idiopathic Pulmonary Fibrosis Network (IPFnet) had proposed in 2007 the following definition of an acute exacerbation of IPF [3]:

- A previous or concurrent diagnosis of IPF.
- Deterioration within 30 days.
- New bilateral ground glass opacities and/or consolidation on a background of reticular or honeycomb pattern consistent with usual interstitial pneumonia (UIP).
- No evidence of pulmonary infection by endotracheal aspiration or bronchoalveolar lavage.
- Exclusion of alternative causes, including left heart failure, pulmonary embolism, or an identifiable cause of acute lung injury.

The above-mentioned criteria were revised in 2016 [4].

AE-IPF is defined as an acute, clinically significant respiratory deterioration characterized by evidence of new widespread alveolar abnormality. The revised diagnostic criteria are [4]:

- Previous or concurrent diagnosis of IPF.
- Acute worsening or development of dyspnea, typically less than one-month duration.
- Computed tomography with new bilateral ground-glass opacity and/or consolidation superimposed on a background pattern consistent with the usual interstitial pneumonia pattern.
- Deterioration not fully explained by cardiac failure or fluid overload.

The exact causes of AE-ILDs are thought to be related to a variety of factors, including viral infections, environmental exposures such as air pollution, drug toxicity, procedures such as bronchoscopy or VATS, and microaspiration. Regarding the effect of smoking as a risk factor for acute exacerbation of IPF, Song et al. reported that never-smoking status and low FVC were risk factors for AE-IPF [5]. In the study of Mura, however, concomitant emphysema was a risk factor for AE-IPF [6]. Collard et al. examined the risk factors for AE-IPF in the prospective INPULSIS study. They showed that cigarette smoking was associated with increased risk for AE [7]. When an identifiable factor is found, the AE-ILD is characterized as extrinsic-triggered. A number of intrinsic factors have also been identified in patients with AE-IPF, including impaired epithelial homeostasis, immune polarization in macrophages, and possibly autoimmunity against heat-shock proteins. AE-ILD is considered idiopathic when there is no identifiable extrinsic trigger [4].

Among patients with all forms of ILD with acute exacerbations, 52% of admissions for decline of respiratory function were idiopathic, 20% due to infection, 15% due to subacute progression or end-stage disease, 6% due to heart failure or severe pulmonary hypertension, 4% due to venous thromboembolism, and 2% due to diffuse alveolar hemorrhage or perioperative exacerbation [8].

Further research is necessary to understand the causes of AE-ILDs better and to develop more effective treatments for these exacerbations. Treatment options for AE-ILDs include oxygen therapy, corticosteroids, and antibiotics, but there are no targeted treatments that have been proven to be effective in treating these patients [9].

Diagnosis of AE-ILD can be challenging. The clinical condition of these patients is so severely impaired due to the disease exacerbation that even minimally invasive diagnostic procedures, such as bronchoscopy with bronchoalveolar lavage, can have detrimental effects on the clinical course of the patients. Moreover, invasive lung biopsies are associated with increased procedure-associated mortality, which often makes them an unacceptable diagnostic tool in the clinical setting of an AE-ILD [4,9]. Hence, it is essential to find reliable biological markers that can be used non-invasively to confirm the diagnosis of AE-LD and perhaps provide prognostic information.

A biomarker is a biological molecule or genetic feature that can be used to identify the presence of a disease or condition. Biomarkers can be used to diagnose, monitor, and evaluate treatment outcomes for a variety of diseases, including interstitial lung diseases [10,11]. Ideally, biomarkers should have an additive value to other well-established clinical or functional disease markers and should be able to substitute for invasive diagnostic or prognostic procedures.

The focus of the present review will be placed on the use of biomarkers for the diagnosis and prognosis of patients with AE-ILDs.

## 2. Biomarkers for Acute Exacerbations of IPF

Currently, there are no established biomarkers in clinical practice that can predict the onset or outcome of AE-IPF. However, several biomarkers have been proposed as potential predictors of AE-IPF.

### 2.1. Biomarkers Associated with Alveolar Epithelial Cell Dysfunction

The Krebs von den Lungen 6 (KL-6) glycoprotein is a mucin-like, high-molecular-weight protein expressed on the surface membrane of alveolar epithelial cells. Proliferating, stimulated, or injured alveolar epithelial cells release KL-6 into blood vessels. Increased levels of KL-6 in the serum have been found in patients with ILDs [12]. It has also been shown that high levels of KL-6 are associated with AE-IPF. In a prospective study of 77 patients with IPF, the correlation between baseline serum levels of KL-6 and the incidence of AE was evaluated; thirteen patients (17%) experienced AE during follow-up and baseline serum KL-6 levels were significantly higher in patients who developed AE than in patients with stable IPF. At a cut-off level of 1300 U/mL for KL-6, the sensitivity, specificity, accuracy, and likelihood ratio to predict AE were 92%, 61%, 66%, and 2.36, respectively. In the Kaplan–Meier analysis, patients with baseline serum KL-6 level >/= 1300 U/mL experienced earlier onset of AE (*p* = 0.002). In the multivariate analysis, baseline serum KL-6 (both continuous and at a cut-off level of >/= 1300 U/mL) was an independent predictive factor for AE-IPF [13]. Choi et al. prospectively searched for biomarkers associated with AE-IPF in 96 IPF patients. High C-reactive protein levels and an increase of more than 10% in KL-6 blood levels in a week could predict mortality [14].

In another study, IPF patients with increased serum KL-6 levels during follow-up had a significantly steeper decline in FVC than those without increased KL-6 (−201 vs. −50.7 mL/year; *p* = 0.0001). Patients with both initial serum KL-6 >/= 1000 U/mL and serial increase in serum KL-6 had the steepest decline, suggesting a role for KL-6 predicting a worse lung functional status [15], and thus indirectly associated with increased risk of exacerbations, since reduced lung function is a risk factor for AE-IPF. Another study identified increased KL-6 serum levels as a risk factor for AE-IPF in patients treated with nintedanib [16]. Baseline KL-6 levels, as well as serial changes of KL-6, were associated with progressive disease (including AE) in a cohort of 188 patients under antifibrotic treatment [17]. Despite these positive results, a meta-analysis of fourteen studies regarding the association of KL-6 levels with AE-IPF showed that the detection power of the biomarker is limited, and no relationship between biomarker concentrations and mortality was found. The authors suggested caution when extending obtained results to non-Asian populations [18]. Nevertheless, a significant number of specialized and non-specialized ILD institutions reported using KL-6 levels in the assessment of patients with AE-IPF (24% and 15%, respectively) [19].

Surfactant proteins (SPs) reduce alveolar surface tension and prevent lung collapse. They are synthesized and secreted by alveolar epithelial cells (AEC) II. Patients with IPF, as well as those with other ILDs, have significantly elevated levels of SP-D and SP-A in their serum and BALF [20,21].

Increased levels of SP-D were observed in patients with AE-IPF compared to those with stable disease [20]. In a meta-analysis of a total of 1289 IPF patients, SP-A levels could better differentiate between IPF and other ILDs in stable disease, and higher SP-A and SP-D levels were associated with a significantly higher risk of death. Moreover, elevated SP-A and SP-D indicated AE-IPF [22]. In a retrospective study of patients with AE of idiopathic interstitial pneumonias (with the most part consisting of patients with AE-IPF), high SP-D serum levels and a diffuse HRCT pattern of lung involvement at the time of AE was associated with worse survival. The authors suggested that increased SP-D levels at baseline are a marker of an increased number of hyperplastic AECs, which makes them more vulnerable to various injuries that can induce an AE [23].

In the normal process of cell replication, telomeres protect chromosome ends from progressive shortening [24]. Abnormal telomere shortening has been found in patients with familiar pulmonary fibrosis and patients with IPF [25,26,27]. In a retrospective study of patients with IPF and other ILDs, short telomere length was associated with AE-IPF and was an independent risk factor for mortality [28].

Growth differentiation factor 15 (GDF-15), also named macrophage inhibitory cytokine-1 (MIC-1), belongs to the transforming growth factor-β (TGF-β) superfamily. There is an increase in the expression and upregulation of GDF-15 in epithelial cells in IPF. GDF-15 is a useful biomarker for epithelial stress associated with poor IPF outcomes. In a study of 47 patients with AE-IPF and 61 patients with stable IPF, serum GDF-15 levels, as well as the protein and mRNA expressions of GDF-15 in the lung, were significantly elevated in AE-IPF patients compared with stable IPF cases. A serum GDF-15 level above 989.3 pg/mL was a risk factor for AE occurrence, and the level above 1075.76 pg/mL was an independent predictor for survival in IPF cases [29].

### 2.2. Biomarkers Associated with Extracellular Matrix Remodeling and Fibroproliferation

Matrix metalloproteinases (MMPs) are endopeptidases with zinc in their structure that degrade proteins of the extracellular matrix. Development, morphogenesis, tissue repair, and remodeling rely heavily on the timely degradation of extracellular matrix. By regulating extracellular matrix turnover, MMPs contribute to the pathogenesis of fibrosis [30,31,32]. MMP-7 and MMP-1 are the most extensively studied MMPs in patients with IPF. There was an increase in MMP-7 and MMP-1 serum levels in patients with IPF compared with those with non-IPF ILDs [33], and MMP-7, osteopontin, and SP-D together increase the diagnostic accuracy of a biomarker index that distinguishes patients with IPF from those without [21]. MMP-7 levels are associated with mortality and disease progression in IPF, as it was shown in a biomarker analysis of the BUILD-3 trial [34]. A clinical and molecular mortality prediction index, which included gender, forced vital capacity (FVC), diffusing capacity for carbon monoxide (DLCO), and MMP-7 plasma levels, predicted poor overall survival and poor transplant-free survival among patients with IPF [35]. An analysis of a large clinical trial cohort, including clinical covariates and *mucin 5B* (*MUC5B*) genotype, revealed an independent association between MMP-7 and survival in IPF patients [36]. It has been difficult, however, to integrate MMP-7 into clinical practice due to the lack of reproducible and uniform cut-off points between studies, and a direct association between MMPs and the risk of AE-IPF has not been established yet.

Biomarkers released by matrix metalloproteinase-mediated degradation of collagen have also been examined in AE-IPF; such a degradation product, versican, was found to be associated with mortality in a study of 68 patients with AE of idiopathic interstitial pneumonias, including 28 patients with AE-IPF [37].

A disintegrin and metalloproteinases (ADAMs) are believed to be involved in the pathogenesis of many fibrosis-related diseases. ADAM-17 was increased in IPF and other ILDs, such as CTD-ILD [38]. ADAM-17 has been reported to be increased in patients with AE-IPF compared to stable IPF [38].

Human epididymis protein 4 (HE4) is widely used as a biomarker in ovarian and endometrial cancer. HE4 inhibits the function of various proteases, such as MMPs and serine proteases, suppresses the degradation of type I collagen, and plays a crucial role in the pathogenesis of fibrosis. In a retrospective study of 59 patients with stable IPF and AE-IPF, the expression of serum HE4 was elevated, especially in AE-IPF patients, as was KL-6. In the multivariate analysis, HE4 levels, as well as GAP index, were associated with survival. Histologically, HE4 could be found in the mucosal epithelium of dilated bronchi [39].

The levels of Lysyl oxidase-like proteins (LOXL) have also been associated with increased mortality and progression of IPF; however, a direct association with AE-IPF has not been shown [40].

Circulating fibrocytes are progenitors of fibroblasts and myofibroblasts. IPF patients with stable disease have been shown to have increased circulating fibrocytes [41], and patients with acute exacerbations had even higher levels. Fibrocyte numbers did not correlate with lung function or radiologic severity scores, but they were an independent predictor of early mortality. The mean survival of patients with fibrocytes higher than 5% of total blood leukocytes was 7.5 months compared with 27 months for patients with less than 5% [42].

Non-structural extra-cellular matrix protein, periostin, promotes mesenchymal cell proliferation and fibrosis through its expression at sites of injury or inflammation. In a recent study, serum monomeric periostin levels were prospectively measured from onset of AE in 37 patients with AE of chronic interstitial pneumonia to day 14, and its association with outcome was evaluated. Serum monomeric periostin levels were significantly higher in patients with AE compared to the ones with stable IPF and decreased significantly in AE survivors compared to AE non-survivors. Furthermore, a decrease of serum monomeric periostin levels in patients with AE was associated with better survival in 3 months after AE (OR 12.4) [43]. Immunohistochemical analysis of the lungs of patients with AE of familial lung fibrosis has shown marked accumulation of periostin in the active fibrotic lesions, whereas intact and burned-out areas did not show significant expression of periostin [44].

An important cytokine involved in tissue repair is osteopontin (OPN). It has been shown that OPN can activate MMP-7 and stimulate fibroblast migration and proliferation [45]. IPF patients had elevated serum OPN levels, especially in AE-IPF, compared with healthy controls. The enhanced expression of OPN was also found in alveolar epithelial cells and alveolar macrophages in lung tissue from IPF patients. Compared with stable IPF, serum OPN levels in AE-IPF were significantly increased, correlated well with inflammatory markers such as CRP and LDH, and were associated with poor outcome [46].

### 2.3. Biomarkers Associated with Immune Dysfunction

The innate immune response to infection and tissue injury is mediated by toll-like receptors (TLRs) [47]. IPF patients with L412F polymorphism of TLR3 produce altered cytokines and exhibit dysregulated fibroblast proliferation. Clinical progression and increased mortality were associated with this polymorphism in patients with IPF [48]. In a recently published study, patients with L412F-variant IPF were significantly more likely to die from AEs, and L412F-heterozygous IPF lung fibroblasts had reduced antibacterial TLR responses to various stimuli, including LPS (TLR4), Pam3CYSK4 (TLR1/2), flagellin (TLR5), and FSL-1 (TLR6/1), and to live Pseudomonas aeruginosa infection. Based on 16S RNA sequencing, the authors demonstrated that patients with IPF who are heterozygous for L412F have a dysregulated lung microbiome with increased Streptococcus and Staphylococcus spp. populations, suggesting a candidate role for *TLR3* L412F in viral- and bacterial-mediated AE death [49].

The toll-interacting protein (TOLLIP) is an inhibitor of the toll-like receptors (TLRs) 2 and 4, which are active in the lung, thereby suppressing tumor necrosis factor-a (TNF-a) and interleukin 6 (IL-6) production. Genetic variants of *TOLLIP* have also been reported to be associated with the development and/or prognosis of IPF [50]. In IPF patients, minor allele *TOLLIP rs5743890* was associated with worse survival and a more rapid progression of IPF, which could be useful for stratifying IPF patients at baseline [51]. Moreover, a tendency for a protective role for AE of the minor allele (T) was observed [51].

Defensins are proteins secreted by neutrophils which exert antimicrobial activity [52]. Acute exacerbations of IPF have been associated with increased plasma defensins, but their levels were not specific enough to serve as biomarkers [53].

C-X-C motif chemokine ligand 13 (CXCL13) facilitates B-cell trafficking in lymphoid aggregates and inflammatory foci. The presence of elevated CXCL13 protein levels in patients with IPF has been linked to increased mortality, and CXCL13 levels were highest in IPF patients with AE-IPF [54].

S 100 calcium binding proteins 8 and 9 (S100A8 and S100A9) are released mainly by activated neutrophils, which promote inflammation and decrease vascular endothelial cell integrity, consequently inducing further neutrophil extravasation. In ILDs, S100A8 and S100A9 are more often produced in patients with IPF with chronic-phase disease than in patients with other diseases, such as sarcoidosis or CTD-ILD. In a retrospective study of 37 patients with AE, patients with higher levels of S100A8 or S100A9 showed significantly worse 3-month survival than those with lower levels. In the multivariate analysis, the serum levels of both S100A8 and S100A9 were significant prognostic factors of AE-IPF [55]. In another study, serum S100A4 had a high predictive value for postoperative AE of IPF and short-term mortality after lung resection [56].

Immune cells, particularly macrophages and lymphocytes, have been implicated in the pathogenesis of ILDs. CD4+ cells in IPF are in a highly activated status [57]. Decreased serum CD28+ CD4+ T cells, as well as increased CD8+ T-cell numbers in lung biopsies from patients with IPF, have been associated with prognosis and disease severity [58,59]. In a recent small study of mass cytometry in BALF of patients with ILDs, including a patient with AE-IPF, different monocyte, B-cell, and T-cell populations were found in patients with IPF compared to CTD-ILD. The patient with AE-IPF exhibited a CD^14^ CD36^hi^ CD84^hi^ CCR2^−^ monocyte population. The same phenotype was found in patients with progressive IPF [60].

### 2.4. Inflammatory and Anti-Inflammatory Cytokines

Increased levels of circulating pro-inflammatory cytokines have been reported during AE-IPF. Interleukin 1b (IL-1b) upregulates interleukin 8 (IL-8), which is critical for neutrophil recruitment. IL-8 is produced by phagocytes. Higher concentrations of IL-8 have been found in the BALF of patients with IPF and fibrosis associated with collagen vascular diseases [61]. Patients with IPF exhibit higher levels of IL-8 compared to patients with fibrosis due to rheumatic disease. Moreover, IL-8 levels correlate with BALF neutrophilia. IL-8 serum levels have also been shown to be high in patients with IPF and to correlate with BALF IL-8 levels and BALF neutrophilia [62]. Increased IL-8 levels were associated with worse lung function [62], and high concentrations of plasma IL-8 levels also reflected poor overall survival, transplant-free survival, and disease progression-free survival [35]. Most important, blood IL-8 levels were higher in patients with AE-IPF, and an increase by 1 pg/mL increased the odds of death by 6.7% [63].

IL-1b levels have been reported to be higher in patients with AE-IPF than in stable IPF [64]. Increased serum IL-1b levels (>5 pg/mL) were an independent risk factor for 3-month mortality in patients with AE-IPF [65]. Hemoperfusion with polymyxin has been tried as a therapeutic option in patients with AE-IPF. In such patients, IL-1b was significantly absorbed from the polymyxin fibers, a result associated with better oxygenation of patients with AE-IPF in a small study [66].

Interleukin 6 (IL-6) is a pro-inflammatory, pro-fibrotic cytokine. Increased serum levels of IL-6 above 25.20 pg/mL have been reported to be diagnostic of AE-IPF and to be an independent risk factor for mortality [67]. An increase by 1 pg/mL in the blood levels of IL-6 increased the odds of death by 5.6% in patients with AE-IPF [63].

Interleukin 35 (IL-35) is a cytokine which is considered to have anti-fibrotic and anti-inflammatory effects by affecting interleukin 17 (IL-17) production. IL-35 also prevents the phosphorylation of Smad3, a downstream cascade of transforming growth factor (TGF-b) signaling, thus blocking TGF-b binding to its receptor and preventing the differentiation of T helper 17 (Th17) cells [68]. In a recent study of cytokines in BALF of patients with ILDs of various etiologies (fibrotic and non-fibrotic), the BALF concentration of IL-35 was lower in fibrotic ILDs and negatively associated with BALF concentrations of TGF-b. Moreover, an increased TGF-b/IL-35 ratio was associated with fibrotic ILDs, was highest in patients with IPF, and correlated to worse lung function, as measured by DLCO. Low TGF-b/IL-17 ratio was also associated with worse survival (4.2 vs. 5.5 years, respectively) [68].

### 2.5. Microbiome

Lungs have been traditionally considered sterile of bacteria. In part, this notion was due to the inability of traditional culture techniques to isolate bacteria from lung specimens. The introduction of sequencing of the 16S rRNA gene technique allowed recognition of the fact that bacteria not only exist within the human lung, but they are also altered in lung disease. A higher microbial burden is associated with a worse prognosis in stable IPF. It has been shown that patients with AE-IPF have a higher microbial burden in their lungs, which has been associated with higher morbidity and mortality rates. A significant increase was noticed for *Proteobacteria*, *Campylobacter* spp., and *Stenotrophomona* spp., while a significant decrease was found in *Veillonella* spp. and *Campylobacter* spp [69]. In sputum cultures of 170 patients with AE-IPF, gram-negative bacteria were found to dominate, consisting of 89% of the 38 different strains that have been found. *Klebsiella pneumonia* accounted for 26%, *Mycobacteria tuberculosis* for 21%, and *Acinetobacter baumannii* for 10% of the total strains [70]. Although lung microbiome has been associated with disease severity, progression risk, AE-IPF, and mortality, causation has yet to be established [71,72].

### 2.6. Non-Coding RNAs

Non-coding RNAs represent a type of epigenetic change, i.e., a gene expression change that does not involve a change in the DNA [73]. Long non-coding RNAs (lncRNAs) and micro-RNAs (mi-RNAs) represent the two major types of non-coding RNA. In a study of patients with IPF, six mi-RNAs showed differentiated expression between AE-IPF and stable IPF patients. In the validation cohort, *miR-25-3p* and *let-7d-5p* in plasma were differentially expressed between AE-IPF and stable IPF, suggesting that a combination of these two mi-RNAs may be a potential biomarker for AE-IPF from IPF [74].

In a study, the *miR-302c*, *miR-423*, *miR-210*, *miR-376C*, and *miR-185 mi-RNAs* were found to be related to the severity of IPF, differentiating fast and slow progressors [75].

### 2.7. Mitochondrial DNA

In aging-related lung diseases, such as IPF, mitochondrial dysfunction is critical in regulating programmed cell death in alveolar epithelial cells [76]. Mitochondrial DNA, released by necrotic cells as well as viable cells after exposure to hazardous stimuli, is a marker of mitochondrial dysfunction; mtDNA was found to be elevated in the BAL of patients with IPF, and it was present in high concentrations in the lungs and blood of patients with IPF, as shown in two independent IPF Cohorts [77]. More significantly, high mtDNA levels were associated with a bad prognosis and progressive disease and could independently predict survival [77].

### 2.8. Coagulation Factors

Histologically, AE-IPF is characterized by diffuse alveolar damage (DAD) overlying IPF fibrosis. AE-IPF was associated with alveolar hemorrhage in ca. 25% of the patients and with pulmonary thromboembolism in ca. 20%, suggesting that capillary injury and thrombosis play a significant role in AE-IPF [78]. A study by Collard et al. examined the plasma profile of coagulant factors in AE-IPF patients; proteins C, thrombomodulin, and plasminogen activator inhibitor 1 (PAI-1) levels were higher than in stable IPF patients. Patients with AE-IPF also had higher plasma levels of fibrinogen degradation products (FDP), d-dimer, and thrombin–antithrombin complex compared to those with IPF and pneumonia. On day seven after the onset of AE-IPF, survivors had significantly lower serum thrombin–antithrombin complex levels than non-survivors [20]. A significant increase in d-dimer and thrombomodulin was also found in BALF of patients with AE-IPF compared to patients with stable IPF [79]. It appears that AE-IPF is associated with both endothelial injury and coagulopathy. Thrombomodulin, in particular, was tried not only as a biomarker but also as a therapeutic means for patients with AE-IPF; despite initial promising results, a randomized, placebo-controlled phase III study of recombinant human thrombomodulin failed to show a survival benefit in patients with AE-IPF compared to placebo [80].

### 2.9. Blood Cell Count Derived Inflammation Indexes

#### 2.9.1. Neutrophil-to-Lymphocyte Ratio (NLR)

NLR is a ratio calculated from the absolute number of neutrophils and lymphocytes in the blood. Elevated NLR has been found in IPF patients with acute exacerbation, and it has been proposed that measuring NLR could be used to predict AE-IPF. In a multicenter study using discovery and validation cohorts, high values of NLR (>/=2.9 vs. <2.9) were associated with an increased risk of mortality in IPF (HR 2.04). This was confirmed in the validation cohort. NLR correlated with GAP stage and GAP index, which are widely used scores combining gender, age, and pulmonary function to predict survival in IPF [81]. These results confirm the results of previous studies [82]. A study of 278 patients with IPF showed that NLR can effectively predict AE-IPF; in 116 acute exacerbation IPF patients, the results of the Cox multiple regression model also indicated that the NLR was a significant prognostic factor (OR = 1.022, 95% CI 1.001–1.044, *p* = 0.036). The NLR before death was also significantly higher than that at admission in patients with AE-IPF who did not survive (*p* = 0.014) [83]. It is not completely clear why NLR depicts a worse prognosis in IPF. It is suggested that NLR is a marker of ongoing inflammation. It is known that neutrophilia in the BALF of patients with IPF correlates with poor outcome. BALF neutrophilia is associated with both increased microbiome burden and progressive IPF, with subtle changes in the microbiome implicated in the initiation and progression of IPF in the absence of identified infection [84]. Another hypothesis is that the lung may orchestrate the disposal of aged neutrophils by targeting them for recirculation to and disposal in the bone marrow. The neutrophil count, however, is not as strong a predictor of mortality in IPF as NLR, suggesting that both neutrophil activation and lymphocyte exhaustion may be relevant [81]. NLR is a cheap, readily available marker, making it particularly attractive for use in clinical practice.

#### 2.9.2. Platelet-to-Lymphocyte Ratio (PLR)

Apart from NLR, other blood cell ratios have been shown to be associated with prognosis in IPF. Such a marker is the PLR [85]. In a post hoc analysis of the placebo-treated IPF patients, who participated in the pirfenidone studies ASCEND and CAPACITY, the highest PLR (along with NLR) changes during the study were associated with worse outcomes [82]. Other studies, however, have not confirmed these results [83,86].

#### 2.9.3. The Monocyte to Lymphocyte Ratio (MLR)

Systemic inflammation response index (SIRI) and aggregate index of systemic inflammation (AISI) as indexes of inflammation have also been reported to correlate with IPF and worse functional status [85].

### 2.10. Multiple Biomarker Signatures

Multiple biomarker signatures can be used more effectively than a single protein in the diagnosis and prognosis of IPF. The combination of serum MMP-1 and MMP-7 levels, for example, provided higher positive and negative predictive values for the diagnosis of IPF than either protein on its own [87]. Serum biomarkers cluster of differentiation 28 (CD28), inducible T-cell co-stimulator (ICOS), LCK- protooncogene (*LCK*), IL-2 inducible T-cell kinase (*ITK*) as part of a 52-gene RNA signature [88], a composite score of OPN, periostin, MMP-7 and intercellular adhesion molecule 1 (ICAM-1) [89], and the Total-to-Background Ratio (TBR) calculated from 18F-FDG-PET imaging [90] have been reported to effectively differentiate low from high risk patients with IPF. In an RNA extraction study of patients with AE-IPF, five hundred and seventy-nine genes were differentially expressed between stable IPF and AE-IPF. Cyclin A2 (CCNA2) and alpha-defensins were among the most upregulated genes. CCNA2 and alpha-defensin protein levels were also higher and localized to the epithelium of IPF-AE, where widespread apoptosis was also detected [91].

PaO_2_/FiO_2_ ratio, diffuse HRCT pattern, and serum C-reactive protein (CRP) were significantly associated with 3-month mortality in patients with AE-IPF [92]. In another study, soluble intercellular adhesion molecule 1 (sICAM-1), macrophage migration inhibitor factor (MIF), IL-1b., and soluble urokinase plasminogen activator receptor (su-PAR) were all increased in patients with AE-IPF and MIF, and IL-1b, in particular, was independently associated with 3-month mortality [65].

Proteomic comparative analysis of BALF in stable IPF patients vs. AE-IPF revealed that proteins involved in the propagation of β-catenin WNT transduction signal, proteins upregulated in lung carcinogenesis (IGKC, S100A9, PEDF, IGHG1, ALDOA, A1AT, HPT, CO3, and PIGR), as well as acute phase proteins involved in protease–antiprotease imbalance (such as a1 antithrypsin fragments) correlated to AE-IPF [93].

## 3. Biomarkers for Acute Exacerbations of NSIP (AE-NSIP)

One type of idiopathic interstitial pneumonia (IIP) is nonspecific interstitial pneumonia (NSIP). Besides being idiopathic, NSIP can be associated with HIV infection, connective tissue disease, various drugs, and hypersensitivity pneumonitis. In addition, it can occur in combination with other IIPs. The nonspecific nature of NSIP is due to the absence of the histopathologic characteristics of usual interstitial pneumonia (UIP), desquamative interstitial pneumonia (DIP), respiratory bronchiolitis-associated interstitial lung disease (RB-ILD), or acute interstitial pneumonia (AIP) [94]. NSIP is characterized by a homogeneous appearance of dense or loose interstitial fibrosis accompanied by mild or moderate chronic interstitial inflammation and rare or absent fibroblast foci, dense alveolar septal fibrosis, organizing pneumonia, granulomas, lymphocyte or eosinophil infiltration, and temporal heterogeneity, which are characteristic of chronic interstitial pneumonia [94]. Acute exacerbations can occur in the setting of NSIP [95]. Numerous biomarkers have been studied in NSIP, most compared to IPF. However, specific NSIP biomarkers are not used in clinical practice. Persistently high levels of KL-6 and SP-D correlated with progressive disease in idiopathic fibrotic NSIP [96]. Serum levels of KL-6 in NSIP positively correlated with the total HRCT score and overall extent of interstitial disease. Serum levels of SP-D in NSIP showed a positive correlation with the area of ground-glass attenuation without traction bronchiectasis and the inflammatory CT pattern and inversely correlated with the areas of ground-glass attenuation with traction bronchiectasis and the fibrotic CT pattern. The follow-up CT and serum marker changes after treatment showed that the percent change of disease extent was reflected in both markers, especially KL-6 [97]. In the BALF, increased levels of KL-6 and calgranulin B (a small calcium-binding protein with various immunological functions, mainly involved in chronic inflammation) were found in patients with IPF and idiopathic NSIP and correlated with worse functional parameters [98]. Proteomic analysis can also be useful to differentiate UIP from NSIP [99]. Since worsening of lung function and disease progression are associated with the development of AE-ILD, we could consider the previous reported markers as indirect markers of AE-ILD. However, more studies are needed to prove this concept.

## 4. Biomarkers for Acute Exacerbations of Connective Tissue Diseases Interstitial Lung Disease (CTD-ILD)

The development of acute ILD may occur with any CTD, as a de novo acute interstitial pneumonia (AIP) or as an acute exacerbation of an ILD that already exists. Acute exacerbations have been described in rheumatoid arthritis (RA), polymyositis/dermatomyositis (PM/DM), systemic sclerosis (SSc), systemic lupus erythematosus (SLE), and mixed connective tissue disease (MCTD). Common underlying pathology patterns are that of NSIP and UIP [100]. Common histological pattern of an acute exacerbation is that of DAD, as usually seen in ARDS. Cases of organizing pneumonia have also been described [95,101]. The definition of AE-ILD associated with CTD is similar to the one used for AE-IPF [101]. The diagnosis of an AE-ILD in CTD can be particularly challenging, since differential diagnosis includes drug-induced pulmonary toxicity and opportunistic infections, all of which are difficult to exclude based solely on radiographic and clinical findings.

Autoimmune biomarkers are particularly useful for the diagnosis of the underlying CTD in the case of a novo presentation of ILD [102]. However, the sensitivity and specificity of the autoantibodies used in clinical practice vary widely; antinuclear antibodies (ANA), for example, have high sensitivity for SLE, SSc, and RA, but their specificity is very low. On the other hand, anti-RNA polymerase antibodies have a 20% sensitivity but a specificity of 98% for the diagnosis of SSc [11]. In this section, we will report current knowledge regarding biomarkers for the two most common CTD-ILD, i.e., RA-ILD and SSc-ILD.

### 4.1. Rheumatoid Arthritis

As a major extraarticular feature of RA, lung involvement affects 10–60% of patients. The prevalence of ILD ranges from 5 to 58%, with clinically significant RA-ILD occurring in less than 15% of cases. Antibodies to citrullinated protein antibodies (anti-CCPs) and rheumatoid factors (RFs) are frequently found in the serum of patients with RA. RF levels greater than 90 international units/mL may identify patients at higher risk of ILD [103]. It has been shown that specific subtypes of anti-CCP, such as Hsp90 auto-antibodies, are associated with ILD [104]. RA-ILD is associated with the gain-of-function MUC5B promoter variant rs35705950, which is also strongly associated with IPF [105,106]. MUC5B variants are also associated with usual interstitial pneumonia (UIP) on high resolution computed tomography in patients with RA-ILD. There are no reports of ILD associated with this MUC5B variant in the context of systemic sclerosis or inflammatory myositis.

Most of the biomarkers tested in IPF have also been tested in RA-ILD [107]. However, data regarding biomarkers in RA-associated AE-ILD are limited. Markers of pulmonary vasculopathy, such as vascular cell adhesion molecule 1 (VCAM-1), monocyte chemoattractant protein 1 (MCP-1), and asymmetric dimethylarginine (ADMA), have been reported as useful biomarkers of ILD in RA patients and can help in the differential diagnosis of RA-ILD from IPF [108]. Other reported biomarkers include endothelin-1 [109], interleukin 13, 18 [107], 36a and 36γ [110], and HE4 [111]. It appears that patients with RA-ILD have short telomeres compared to other CTD-ILDs, and they have worse outcomes in terms of survival and lung function than the other CTD-ILDs, suggesting that telomere length plays an important prognostic role in this disease [112].

Age, sex, smoking history, positive RF, and anti-CCP, along with a combinatorial signature of serum biomarkers, including MMP-7, pulmonary and activation-regulated chemokine (PARC), and SP-D, could predict RA-ILD [113].

KL-6 and SP-D have been examined in RA patients with ILD or AE-ILD; no significant difference in their levels in stable RA-ILD and AE-ILD or de novo acute ILD has been reported [114]. A reason could be that KL-6 and SP-D levels fluctuate significantly under immunosuppressive treatment without any association with clinical events [115]. Discordant results have also been published: in a study of 33 patients with RA-associated AE-ILD, KL-6 levels at RA-ILD diagnosis, as well as the difference of KL-6 levels at AE-ILD and baseline could independently predict mortality, with an increased risk of 337% [116]. High KL-6 levels by the diagnosis of RA-ILD were associated with a UIP pattern and increased mortality [117]. The combination of KL-6 and B-lines in lung ultrasound could effectively help in the diagnosis and follow-up of patients with RA-ILD [118].

It has been suggested that the metabolites mannosamine, alliin, kynurenine, and 2-hydroxybutyric acid could successfully discriminate acute ILD in the setting of RA from the stable condition [114].

Radiomic analyses have also been reported to predict mortality in RA-ILD, and its use has been proposed as a digital biomarker [119].

### 4.2. Systemic Sclerosis

The reported prevalence of ILD in SSc is 50–60% [96]. ILD is associated with diffuse cutaneous SSc. Anti-topoisomerase I (also known as anti-Scl-70) antibody) is associated with an increased risk for SSc-associated ILD (sensitivity 45%, specificity 81%). ILD is rare in patients with anticentromere antibodies (ACA). Anti-U3 ribonucleoprotein (RNP), anti-U11/U12 RNP, and anti-Th/To autoantibodies have also been associated with an increased risk for ILD [120,121,122].

In a recent meta-analysis, KL-6, SP-D, and IL-8 in blood and BALF were the best biomarkers of SSc-ILD [123]. Patients with SSc-ILD have higher serum SP-D levels than those without [124]. In combination with serum anti-topoisomerases I antibody, serum SP-D value could detect patients with SSc-ILD with 97% sensitivity, 69% specificity, 80% positive predictive value, and 95% negative predictive value. Furthermore, these two biomarkers could help identify three categories of patients regarding the probability of SSc-ILD (mild, moderate, and high risk) [125]. Known for its negative correlation with lung function and positive correlation with radiological impairment or extensive lung fibrosis, KL-6 serum level can assist in assessing the severity of disease [102]; however, more recent studies did not confirm a correlation of serum KL-6 levels with prognosis [125]. The CCL18 chemokine ligand has been implicated in many lung fibrosing diseases, including SSc-ILD. A higher serum level of CCL-18 is associated with a greater impairment of pulmonary function and a five-fold increase in mortality [126,127]. Other biomarkers associated with SSc-ILD and severity of pulmonary involvement are C-C motif chemokine ligand 2 (CCL-2), chitinase 3 like 1 (YKL-40), MMP-7 and MMP-12, connective tissue growth factor (CTGF), and IL-6 [120]. SSc-ILD severity is also positively correlated with blood levels of circulating endothelial progenitor cells, higher Th1/Th2 lymphocyte ratio, and various mi-RNAs, such as miR-155, miR-29a, and miR-142-3p [120], S100A-12 [128], ADAM-17 [38], and HE4 [129]. An increased Neutrophil-to-lymphocyte ratio has been associated with reduced survival not only in IPF but also in patients with SSc-ILD [130].

The above-described biomarkers are predictive of progressive ILD and mortality. Progressive disease is a risk factor for AE. It is unclear whether these biomarkers can also depict the development of AE-ILD in SSc-ILD.

## 5. Biomarkers for Acute Exacerbations of Hypersensitivity Pneumonitis

Hypersensitivity pneumonitis (HP) is an ILD that results from inhalation of organic antigens in susceptible individuals. HP is classified into fibrotic or nonfibrotic [131], and acute exacerbations can occur in the course of hypersensitivity pneumonitis [95]. Through the formation of antigen–antibody complexes, circulating specific IgG antibodies (precipitins) play a role in disease pathogenesis. Currently, both type III and IV hypersensitive reactions are believed to participate in disease pathogenesis [131]. BALF lymphocyte counts have been proposed as a diagnostic criterion for HP, contributing to the differential diagnosis from IPF and other fibrotic ILDs [132]. As compared to acute and subacute forms, chronic HP has a predominance of CD4 lymphocytes and an increased CD4/CD8 ratio in the BALF. However, these markers are inconsistent and significantly vary, making them difficult to use in clinical settings to diagnose or exclude the disease [131,132]. KL-6 levels have been reported to be increased in patients with HP compared to healthy individuals and could differentiate HP from IPF [133]. However, it remains unclear whether fibrotic or non-fibrotic HP exhibit higher levels of KL-6 [134]. In the study by Okamoto et al., KL-6 levels were not only higher in patients with chronic HP but also increased significantly during acute exacerbations of the disease [135]. KL-6 and SP-D have been included in composite criteria for differential diagnosis of HP vs. IPF, and their levels are reduced after antigen avoidance [136,137]. High baseline SP-D levels were associated with worse prognosis [138]. Various cytokines have been reported to contribute to differential diagnosis between HP and IPF, such as C-C motif chemokine ligand 15 (CCL15) [139]. CCL15 was also associated with the prognosis of HP [139]. CCL15 serum levels were higher in HP compared to IPF [139], while CCL2 was lower in HP compared to IPF [140]. Serum CCL17 levels at baseline were an independent predicting factor of the first episode of AE of HP, with a cut-off value of 285 pg/mL. Serum CCL17 levels were increased during AE compared to baseline [141]. Baseline serum YKL-40 levels were reported to be significantly higher in HP patients than in healthy controls [141] but lower than in patients with other ILDs. Baseline BALF YKL-40 levels in HP patients were the highest among ILD patients. In HP patients, serum YKL-40 correlated with the DLCO at baseline and over time, and HP patients whose disease progressed or who died had higher baseline YKL-40 levels than those who remained stable and survived, suggesting that YKL-40 might be a useful prognostic factor for HP [142]. Serum amyloid A levels can also differentiate between HP and IPF [143,144]. In clinical practice, differential diagnosis between HP and sarcoidosis is of particular value; IL-8 and IL-4Ra in BALF have been reported to help in the differential diagnosis between the two diseases [145,146].

Periostin is an established biomarker of Th2 immune response and fibrogenesis. Serum periostin has been associated with AE of HP. In a study of 63 patients with HP, 13 with IPF, and 113 controls, a significant positive correlation was found between periostin levels and serum KL-6 levels, CD4/CD8 ratio in bronchoalveolar lavage fluid, and fibrosis score on HRCT, and a significant negative correlation was found between periostin levels and carbon monoxide diffusing capacity. Periostin concentrations exceeding 92.5 ng/mL or 89.5 ng/mL were significantly associated with a worse prognosis and a higher frequency of acute exacerbations in chronic HP patients. An independent prognostic factor in the multivariate analysis was a high serum periostin level (92.5 ng/mL or higher) [147].

## 6. Biomarkers Associated with AE-ILDs

Small studies have reported several biomarkers that are associated with AE-ILD in mixed ILD populations. Most of the publications included patients with AE-IPF. The examined biomarkers are the following:

### 6.1. KL-6

A meta-analysis of KL-6 in ILDs reported that ILD patients with severe and progressive disease had higher KL-6 levels. The KL-6 level of patients with severe ILD was 703.41 U/mL higher than in mild ILD. The KL-6 level in the progressive ILD group was 325.98 U/mL higher than that in the non-progressive ILD group. In addition, the KL-6 level of patients in AE of ILD was 545.44 U/mL higher than stable ILD. The higher KL-6 levels in ILD patients predicted poor outcomes. The KL-6 level in patients who died due to ILD was 383.53 U/mL higher than in survivors of ILD. The pooled HR (95%CI) of elevated KL-6 level predicting the mortality of ILD was 2.05 [12].

### 6.2. Neutrophil-to-Lymphocyte Ratio (NLR)

Most data are derived from patients with IPF but reduced survival has also been reported in patients with SSc-ILD [130] and dermatomyositis/polymyositis [148].

### 6.3. D-Dimers

In a retrospective study of a mixed population of patients with ILD, elevated D-dimer levels were associated with a ten-fold increase in risk of developing an AE three months after the D-dimer measurement [149].

### 6.4. Interleukin-6 (IL-6)

The soluble mediator IL-6 plays a pleiotropic role in inflammation, immune responses, and fibrosis. In a retrospective study of 83 patients with ILD, high levels of IL-6, along with lower baseline saturations of peripheral oxygen, were independent risk factors for AE. Similar results have been reported in patients with AE-IPF [63,67].

### 6.5. Heparin-Binding Protein (HBP)

HBP was significantly higher in patients with AE-ILD at the early stage compared to patients with ILD at the stable phase, and this increase was found in the serum and BALF. With the remission of the disease, there was a linear trend of progressive decline. HBP identified ILD patients who had co-infections [150].

### 6.6. BALF Populations

BALF lymphocytosis has been associated with a better prognosis, and BALF neutrophilia with worse prognosis, in a study of 71 patients with AE-ILD. BALF lymphocyte and neutrophil count ≥25% and <20%, respectively, predicted favorable survival after AE [151].

### 6.7. Proteomic Biomarkers

In a recently published study, proteomic analysis was performed in a discovery cohort of 385 patients with various ILDs, including CTD-ILDs, HP, and unclassifiable ILDs. The validation cohort comprised 204 patients. A total of 31 biomarkers were associated with progressive fibrosing ILD in the discovery cohort, with 17 maintaining an association in the validation cohort. Validated biomarkers showed a consistent association with progressive fibrosing ILD irrespective of ILD clinical diagnosis. A proteomic signature comprising 12 biomarkers was derived by machine learning, predicting a progressive fibrosing ILD. Use of this biomarker signature showed not only that progressive fibrotic ILDs, irrespective of aetiology, share common pathogenetic mechanisms but also overcame the modest use performance of other single biomarkers used for prediction of the clinical course of ILDs [152].

## 7. Conclusions

Acute exacerbations are detrimental in the clinical course of ILDs. Early diagnosis before the onset of severe respiratory failure and early recognition of the patients who are more susceptible to AE are of paramount importance for therapeutic decisions in those patients. Despite the fact that treatment of AE in most ILDs is supportive and the use of immunosuppressive treatment—at least in AE-IPF—lacks significant therapeutic effect, early recognition of AE could lead to earlier hospitalization, lower clinical threshold for antibiotic therapy, and faster and probably more extensive diagnostic work-up and decisions for palliative treatment in the right clinical setting. Biomarkers can help in this direction, probably in combination with clinical parameters, lung function indices, and HRCT. Equally important is decision-making, when the diagnosis of an ILD must be first made in the setting of an AE and when the patients are in the intermediate care or intensive care station. Decisions regarding mechanical ventilation, ECMO, and transplantation are often based on the underlying pathology pattern of the ILD.

While the understanding of ILD pathogenesis has grown substantially and biomarker research has progressed, the use of biomarkers for AE-ILD in clinical practice remains limited.

In case of a de novo diagnosed ILD presenting as an AE, diagnostic tests that are currently used are autoantibodies, which can help to differentiate between idiopathic ILDs and ILDs associated with collagen vascular diseases, and the precipitating antibodies supporting the diagnosis of hypersensitivity pneumonitis. In certain cases, BALF lymphocytosis might support an intensive immunosuppressive treatment for AE-ILD, contrary to a neutrophilic BALF.

**Figure 1:**
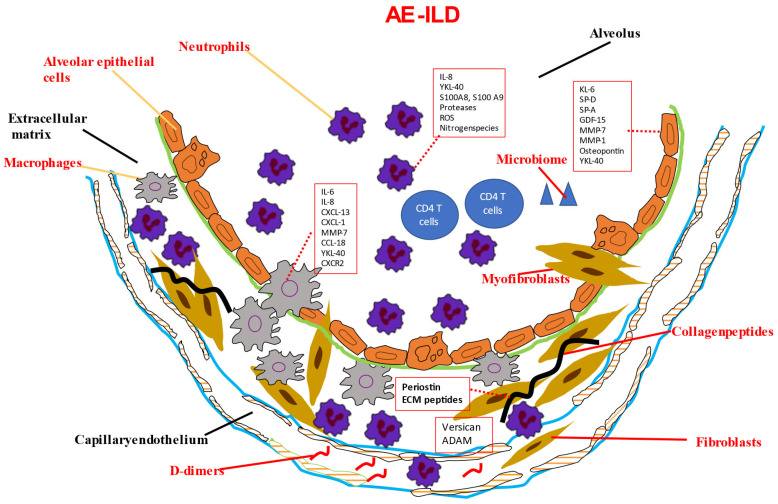
Potential biomarkers in AE-ILD and their cellular source. Due to various stimuli (infections, microaspiration, air pollution, stress, intrinsic acceleration of the underlying fibrosis), injured alveolar epithelial cells and disrupted epithelial barrier lead to the secretion of KL-6, SP-A, and SP-D. Increased KL-6 promotes fibroblast activation through TGF-b signaling. SP-D further aggregates ECM deposition. Matrix metalloproteinase-mediated degradation of collagen leads to the release of versican and ADAM. Periostin is also a marker of increased matrix turnover. During AE-ILDs, lung inflammation is driven by upregulation of macrophage activation pathways. IL-8 and CXCL1 mainly contribute to the neutrophil influx. Neutrophils release numerous proteases and produce reactive oxygen species. Vascular and endothelial damage contributes to the influx of neutrophils.

Despite many biomarkers being studied in AE-ILD (Table 1 and Figure 1), most of these studies are retrospective, with an inevitably low number of patients included (since ILDs are rare and AE-ILDs even more rare). This means that the results of these studies are difficult to implement in everyday praxis, particularly due to the fact that AE-ILDs are not the sole pathogenetic event taking place in a patient; infections, systemic inflammatory reactions, lack of enteral feeding, comorbidities, immunosuppressants, and antibiotics, which are routinely given therapeutically to these patients, reduce the prognostic value of potential biomarkers in AE-ILD. Most biomarkers investigated did not undergo longitudinal analyses, and many were not validated in independent validation cohorts. The use of different study and collection protocols, as well as the use of different biological materials for biomarker analysis, reduces the comparability of different biomarker studies [153,154,155].

KL-6 and MMP-7 are promising and well-studied biomarkers in IPF that might find their way into routine clinical practice in the future. The same applies for simple markers, such as the NLR. However, it seems that multiple biomarker signatures, combined with clinical data, may provide more accurate information regarding AE-ILDs. Simple biomarkers are, however, of particular interest, since they can be used in medium- and low-resource health systems as well.

In prospective clinical trials with large numbers of patients, biomarker analyses in combination with genotyping of these patients may allow the identification of predictors of disease progression and subgroups of patients with varying disease courses [88,152,156].

## Figures and Tables

**Table 1:** Biomarkers reported to be associated with AE-ILD.

ILD Subtype	Biomarker	Compartment	Function	Reference
IPF	KL-6	Serum	Predictive of development of AE-IPF	[12,13,15,16,17]
		Serum	Descriptive of AE-IPF	[14]
		Serum	Predictive of mortality of AE-IPF	[14]
	SP-D	Serum	Descriptive of AE-IPF	[20,22]
		Serum	Predictive of AE-IPF	[22]
		Serum	Predictive of mortality of AE-IPF	[23]
	SP-A	Serum	Descriptive of AE-IPF	[20,22]
		Serum	Predictive of AE-IPF	[22]
	Telomere length	Serum circulating leukocytes	Descriptive of AE-IPF	[28]
	GDF-15	Serum, lung	Descriptive of AE-IPF	[29]
		Serum	Predictive of AE-IPF	[29]
	Versican	Serum	Descriptive of AE-IPF	[37]
		Serum	Predictive of mortality of AE-IPF	[37]
	ADAM-17	Serum	Descriptive of AE-IPF	[38]
	Human epididymis protein 4	Serum	Descriptive of AE-IPF	[39]
	Circulating fibrocytes	Blood	Descriptive of AE-IPF	[42]
	Periostin	Serum	Descriptive of AE-IPF	[43]
		Serum	Predictive of mortality of AE-IPF	[43]
	Osteopontin	Serum	Descriptive of AE-IPF	[46]
		Serum	Predictive of AE-IPF	[46]
	*L412-F*	Blood fibroblasts	Predictive of AE-IPF	[49]
	* TOLLIP rs5743890 *	Blood leukocytes	Predictive of AE-IPF	[51]
	CXCL13	Serum	Descriptive of AE-IPF	[54]
		Serum	Predictive of AE-IPF	[54]
	S100A8 and S100A9	Serum	Predictive of AE-IPF	[55,56]
	IL-8	Serum	Descriptive of AE-IPF	[63]
		Serum	Predictive of mortality of AE-IPF	[63]
	IL-1b	Serum	Descriptive of AE-IPF	[64,65,66]
		Serum	Predictive of mortality of AE-IPF	[65]
	IL-6	Serum	Predictive of AE-IPF	[63,67]
	Lung microbiome changes (increased *Proteobacteria*, *Campylobacter* spp. and *Stenotrophomona* spp., decreased *Veillonella* spp. and *Campylobacter* spp.)	BAL	Descriptive of AE-IPF	[69]
	*miR-25-3p*	Serum	Descriptive of AE-IPF	[74]
	*let-7d-5p*	Serum	Descriptive of AE-IPF	[74]
	D-dimers	Serum	Descriptive of AE-IPF	[20]
	Neutrophil-to-lymphocyte ratio	Blood	Predictive of AE-IPF	[81,82]
		Blood	Predictive of mortality of AE-IPF	[83]
	CCNA2	Lung	Descriptive of AE-IPF	[91]
	sICAM-1, MIF, IL-1b, and su-PAR	Serum	Descriptive of AE-IPF	[65]
		Serum	Predictive of mortality of AE-IPF	[65]
	Proteomic analysis (IGKC, S100A9, PEDF, IGHG1, ALDOA, A1AT, HPT, CO3, and PIGR)	BALF	Descriptive of AE-IPF	[93]
	BALF neutrophilia and lymphopenia	BALF	Predictive of mortality of AE-IPF	[151]
CTD-ILD	D-dimers	Serum	Predictive of AE-ILD	[149]
	IL-6	Serum	Predictive of AE-ILD	[67]
	BALF neutrophilia and lymphopenia	BALF	Predictive of mortality of AE-ILD	[151]
RA-ILD	KL-6	Serum	Prognostic of AE-ILD	[116]
	Mannosamine, Alliin, Kynurenine, 2-hydroxybutyric acid	Serum	Diagnostic of AE-ILD	[114]
HP	KL-6	Serum	Diagnostic of AE-ILD	[135]
	Periostin	Serum	Diagnostic of AE-ILD	[147]
	CCL-17	Serum	Diagnostic of AE-ILD	[141]
		Serum	Predictive of AE-ILD	[141]

## Data Availability

Not applicable.
